# Isoflurane vs. propofol for sedation in invasively ventilated patients with acute hypoxemic respiratory failure: an a priori hypothesis substudy of a randomized controlled trial

**DOI:** 10.1186/s13613-022-01090-w

**Published:** 2022-12-20

**Authors:** Tobias Becher, Andreas Meiser, Ulf Guenther, Martin Bellgardt, Jan Wallenborn, Klaus Kogelmann, Hendrik Bracht, Andreas Falthauser, Jonas Nilsson, Peter Sackey, Patrick Kellner

**Affiliations:** 1 Department of Anesthesiology and Intensive Care Medicine, University Medical Center Schleswig-Holstein, Campus Kiel, Kiel, Germany; 2 Department of Anesthesiology, University Hospital Homburg/Saar, Homburg, Germany; 3 Oldenburg Research Network Emergency- and Intensive Care Medicine (OFNI), University Clinic of Anaesthesiology, Klinikum Oldenburg, Oldenburg, Germany; 4 Department of Anesthesiology, Katholisches Klinikum Bochum, Bochum, Germany; 5 Department of Anesthesiology, HELIOS Klinikum Aue, Aue, Germany; 6 Klinikum Emden, Anaesthesiology, Emden, Germany; 7 Department of Emergency Medicine, University Hospital Ulm, Ulm, Germany; 8 Central Emergency Care Unit and Admission HDU, Wels General Hospital, Wels, Austria; 9 SDS Life Science, Danderyd, Sweden; 10 Department of Physiology and Pharmacology, Unit of Anesthesiology and Intensive Care, Karolinska Institutet, Stockholm, Sweden; 11 Department of Anesthesiology and Intensive Care Medicine, University Medical Center Schleswig-Holstein, Campus Lübeck, Lübeck, Germany

**Keywords:** Inhalation sedation, Isoflurane, Propofol, Acute hypoxemic respiratory failure, Acute respiratory distress syndrome, Ventilator-induced lung injury

## Abstract

**Background:**

Acute hypoxemic respiratory failure (AHRF) is a leading concern in critically ill patients. Experimental and clinical data suggest that early sedation with volatile anesthestics may improve arterial oxygenation and reduce the plasma and alveolar levels of markers of alveolar epithelial injury and of proinflammatory cytokines.

**Methods:**

An a priori hypothesis substudy of a multicenter randomized controlled trial (The Sedaconda trial, EUDRA CT Number 2016-004551-67). In the Sedaconda trial, 301 patients on invasive mechanical ventilation were randomized to 48 h of sedation with isoflurane or propofol in a 1:1 ratio. For the present substudy, patients with a ratio of arterial pressure of oxygen (PaO_2_) to inspired fraction of oxygen (FiO_2_), PaO_2_/FiO_2_, of ≤ 300 mmHg at baseline were included (*n* = 162). The primary endpoint was the change in PaO_2_/FiO_2_ between baseline and the end of study sedation. A subgroup analysis in patients with PaO_2_/FiO_2_ ≤ 200 mmHg was performed (*n* = 82).

**Results:**

Between baseline and the end of study sedation (48 h), oxygenation improved to a similar extent in the isoflurane vs. the propofol group (isoflurane: 199 ± 58 to 219 ± 76 mmHg (*n* = 70), propofol: 202 ± 62 to 236 ± 77 mmHg (*n* = 89); *p* = 0.185). On day seven after randomization, PaO_2_/FiO_2_ was 210 ± 79 mmHg in the isoflurane group (*n* = 41) and 185 ± 87 mmHg in the propofol group (*n* = 44; *p* = 0.411). In the subgroup of patients with PaO_2_/FiO_2_ ≤ 200 mmHg, PaO_2_/FiO_2_ increase between baseline and end of study sedation was 152 ± 33 to 186 ± 54 mmHg for isoflurane (*n* = 37), and 150 ± 38 to 214 ± 85 mmHg for propofol (*n* = 45; *p* = 0.029). On day seven, PaO_2_/FiO_2_ was 198 ± 69 mmHg in patients randomized to isoflurane (*n* = 20) and 174 ± 106 mmHg in patients randomized to propofol (*n* = 20; *p* = 0.933). Both for the whole study population and for the subgroup with PaO_2_/FiO_2_ ≤ 200 mmHg, no significant between-group differences were observed for PaCO_2_, pH and tidal volume as well as 30-day mortality and ventilator-free days alive.

**Conclusions:**

In patients with AHRF, inhaled sedation with isoflurane for a duration of up to 48 h did not lead to improved oxygenation in comparison to intravenous sedation with propofol.

*Trial registration* The main study was registered in the European Medicines Agency’s EU Clinical Trial register (EudraCT), 2016-004551-67, before including the first patient. The present substudy was registered at German Clinical Trials Register (DRKS, ID: DRKS00018959) on January 7th, 2020, before opening the main study data base and obtaining access to study results.

## Background

Long before the onset of the current Covid-19 pandemic, the acute respiratory distress syndrome (ARDS) has been recognized as a major cause of morbidity and mortality in critically ill patients [1, 2]. ARDS is defined as acute hypoxemic respiratory failure (AHRF) of non-cardiac origin with a ratio of arterial partial pressure of oxygen (PaO_2_) to inspired fraction of oxygen (FiO_2_), PaO_2_/FiO_2_ ratio, of less than or equal to 300 mmHg in combination with bilateral opacities on chest X-ray, or CT scan not fully explained by cardiac failure or fluid overload [3]. A recent secondary analysis of the LUNG SAFE study found that AHRF with unilateral opacities or congestive heart failure carries a similar prognosis, even though it does not meet all criteria for ARDS [4].

Since many patients with AHRF require invasive ventilation, some level of sedation may be necessary to establish and maintain endotracheal tube tolerance and to control respiratory drive [5]. According to national and international guidelines, intravenous drugs like propofol, benzodiazepines or alpha-2-agonists are preferably used in this context [6]. As an alternative to intravenous sedatives, the use of volatile anaesthetic agents has gained importance in the recent decades [7, 8].

In addition to their favourable pharmacokinetic properties for sedation, volatile anaesthetics may have particularly favourable effects in patients with or at risk for AHRF, reducing endotoxin-induced lung injury [9, 10] as well as ventilator-induced lung injury [11] and contribute to maintaining the integrity of the alveolar–capillary barrier [12].

In a pilot study, Jabaudon and co-workers randomized 50 patients with moderate-to-severe ARDS to sedation with sevoflurane or midazolam in a 1:1 ratio. 48 h after randomization, they found improved oxygenation and decreased levels of the soluble receptor for advanced glycation end-products (sRAGE), a marker of epithelial injury, with sevoflurane in comparison to midazolam [13].

In the recently published Sedaconda randomized trial [14], 301 patients who received invasive ventilation for various reasons were randomized in a 1:1 ratio to 48 h of sedation with isoflurane or propofol. The Sedaconda trial primarily investigated sedation efficacy and safety, including mechanically ventilated adult patients who were expected to require continuous invasive ventilation and sedation for at least 24 h following randomization and has since then led to European market approval of isoflurane for sedation of patients requiring invasive mechanical ventilation.

ARDS or AHRF were no inclusion criteria in the Sedaconda trial and the influence of volatile anaesthetics on gas exchange and duration of invasive mechanical ventilation in patients with PaO_2_/FiO_2_ ≤ 300 mmHg was not studied. Nevertheless, we expected that a considerable number of patients included in this present study would be suffering from ARDS or AHRF. We therefore conceived a substudy to examine the effects of inhaled sedation with isoflurane on gas exchange and other outcome parameters in patients with PaO_2_/FiO_2_ ≤ 300 mmHg randomized in the Sedaconda trial.

## Methods

An a priori hypothesis substudy of a multicenter randomized controlled trial (the Sedaconda trial, 12). In this trial, patients who had been on invasive mechanical ventilation and sedated with propofol for less than 48 h were randomized to 48 h of sedation with isoflurane or propofol in a 1:1 ratio. For the present substudy, patients with a PaO_2_/FiO_2_ ratio ≤ 300 mmHg at baseline (before randomization) were included.

### Study endpoints

The primary endpoint studied was the change in PaO_2_/FiO_2_ between baseline and the end of study sedation (end ot treatment, EOT, as assessed from the last arterial blood gas (ABG) sample taken before the end of study sedation, usually after 48 h).

Secondary outcome parameters included 30-day mortality, ventilator-free days alive during the first 30 days after randomization, arterial partial pressure of carbon dioxide (PaCO_2_) and pH at end of study sedation, PaO_2_/FiO_2_ (worst value within 24 h) at day seven after randomization, time to extubation in 30-day survivors, the average odds of achieving assisted spontaneous breathing assessed by applied ventilator mode, and the average tidal volume (V_T_) delivered.

A prespecified subgroup analysis was also performed of the predefined outcome parameters in patient with PaO_2_/FiO_2_ of ≤ 200 mmHg.

### Statistical analysis

A partial post hoc power analysis was performed to analyse the power of this substudy for detecting statistical significance of a clinically relevant difference in PaO_2_/FiO_2_ ratio of 40 mmHg between isoflurane and propofol (similar to the difference detected by Jabaudon et al.), using the actually observed pooled PaO_2_/FiO_2_ ratio standard deviation and treatment group sizes of this substudy.

Numerical results are presented as mean ± standard deviation unless specified otherwise.

For numerical endpoints presented in the manuscript, change from baseline was analysed by selected key visits using mixed-effects models with treatment group as fixed effect and centre/pseudo-centre as a random effect. The Sedaconda study was randomized by centre and as for the study report, the centre/pseudo-centre effect were included in analysis models used for this manuscript. Pseudo-centres were established to avoid small strata in the analyses. This was managed by merging sites with less than ten patients into pseudo-centres, by site type. The site types were defined prior to database lock. Site types in the study were: Neurological ICU, Surgery ICU, Internal medicine ICU and General or other. For analysing the primary endpoint, our substudy solely focused on delta between baseline and end of study sedation, not taking into account other timepoints in between. If one PaO_2_/FiO_2_ ratio at end of study sedation was missing, we used the next available value within a time frame of ± 12 h. If PaO_2_/FiO_2_ ratio was not available at end of study sedation ± 12 h, the patient was excluded from the substudy.

Mortality and time to extubation were analysed using the log-rank tests and the comparisons are presented using Kaplan–Meier curves.

Proportion of ventilator-free days alive during the 30 days after randomization was analysed using a mixed-effects model with treatment group as fixed effect and centre/pseudo-centre as a random effect.

Assisted spontaneous breathing was analysed in terms of the odds for a patient achieving the event, assessed from applied ventilator mode.

To assess the on average advantage of isoflurane over propofol, regarding the likelihood of spontaneous breathing across repeated measurements, a generalized linear mixed-effects model (GLMM) with a logistic link function was used.

In the GLMM, the repeated dichotomous assessments (Spontaneous breathing: Yes/No) were modelled using the following explanatory variables: treatment group, timepoint (every 4 h) and timepoint*treatment group interaction. Pseudo-centre was added to the model as a random effect.

An autoregressive covariance structure was used to model the within-subject error.

## Results

**Fig. 1:**
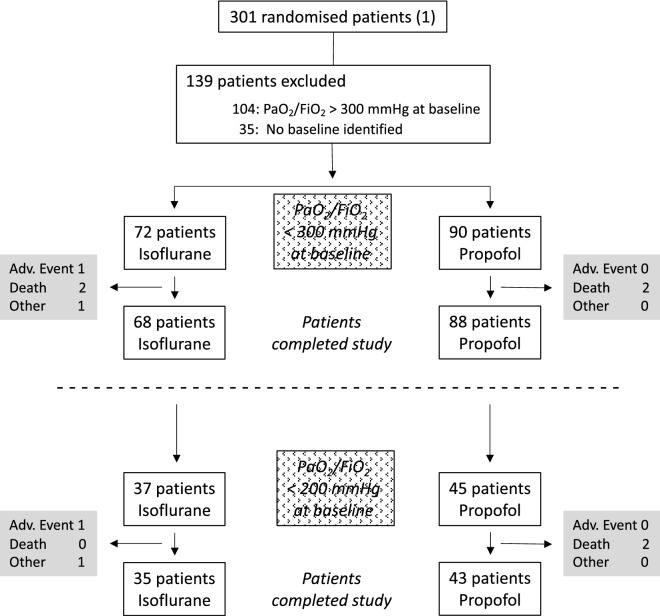
CONSORT diagram. PaO_2_ = arterial partial pressure of oxygen; FiO_2_ = inspired fraction of oxygen

Out of 301 patients who had been randomized in the Sedaconda trial [12], a total of 162 patients (72 in the isoflurane group, 90 in the propofol group) fulfilled the criterion of PaO_2_/FiO_2_ ≤ 300 mmHg at baseline and were thus included in the present substudy. Baseline patient characteristics were balanced between study groups (Table 1). 82 patients had a PaO_2_/FiO_2_ ratio ≤ 200 mmHg (37 in the isoflurane group, 45 in the propofol group). The average duration of study treatment was 38 ± 12 h. Patient flow is depicted in Fig. 1.

**Table 1:** Patient demographics

Variable	Category	Isoflurane (*n* = 72)	Propofol (*n* = 90)	Total (*n* = 162)	*p *(isoflurane vs. propofol)
Age (yrs)	Mean (± SD)	66 (± 11)	65 (± 12)	65.5 (± 12)	0.585
	Min/Max	32/87	33/92	32/92	
Sex	F	19 (26.4%)	29 (32.2%)	48 (29.6%)	n.a
	M	53 (73.6%)	61 (67.8%)	114 (70.4%)	
Height (cm)	Mean (± SD)	174 (± 9)	173 (± 9)	174 (± 9)	1.000
	Min/Max	148/200	155/198	148/200	
Weight (kg)	Mean (± SD)	89 (± 22)	88 (± 24)	88 (± 23)	0.785
	Min/Max	50/148	52/170	50/170	
BMI (kg/m^2^)	Mean (± SD)	29.2 (± 6.9)	29.3 (± 8.0)	29.3 (± 7.5)	0.933
	Min/max	15.4/55.7	16.9/63.2	15.4/63.2	
SAPS II	Mean	47 ± 14 (n = 58)	45 ± 18 (n = 71)	46 ± 16 (n = 129)	0.490
	Min/max	14/86	13/88	13/88	
SOFA	Mean (± SD)	10.6 (± 3.4)	10.3 (± 3.3)	10.4 (± 3.3)	0.571
	Min/max	2/18	2/16	2/18	
PaO_2_/FiO_2_ (mmHg)	Mean (± SD)	199 (± 58)	202 (± 62)	200.5 (± 60)	0.753
	Min/max	84/298	70/298	70/298	
Main Reason for ICU Admission	Medical Neurosurgical	35 (48.6%) 1 (1.4%)	43 (47.8%) 0	78 (48.1%) 1 (0.6%)	n.a
	Surgical Trauma	33 (45.8%) 3 (4.2%)	41 (45.6%) 6 (6.7%)	74 (45.7%) 9 (5.6%)	

*BMI* body mass index, *SAPS II* Simplified Acute Physiology Score, *SOFA* Sequential Organ Failure Assessment, *N.a.* not available

Oxygenation from baseline to the end of study sedation improved to a similar extent in the isoflurane vs. the propofol group and was 20 vs. 34 mmHg (*p* = 0.185; isoflurane: 199 ± 58 to 219 ± 76 mmHg, propofol: 202 ± 62 to 236 ± 77 mmHg). On day seven after randomization, oxygenation was still similar (isoflurane: 210 ± 79 mmHg vs. propofol: 185 ± 87 mmHg; *p* = 0.411).

The partial post hoc power analysis resulted in an estimated power for detecting a clinically relevant difference of 40 mmHg in PaO_2_/FiO_2_ ratio between isoflurane and propofol of 90.1%.

**Fig. 2:**
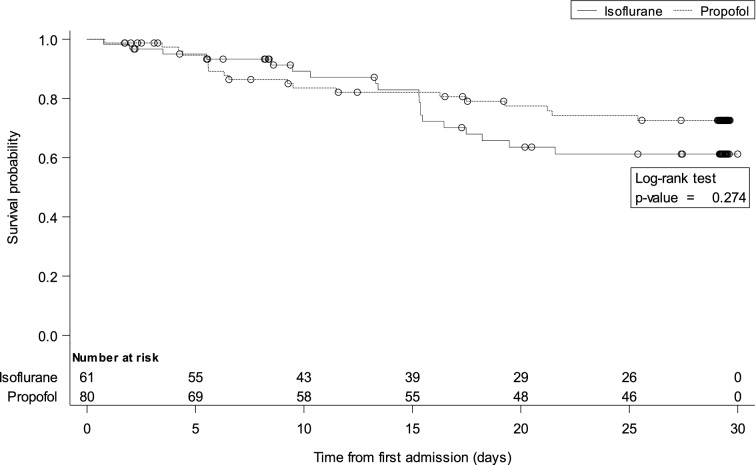
Survival time during the first 30 days after randomization

**Fig. 3:**
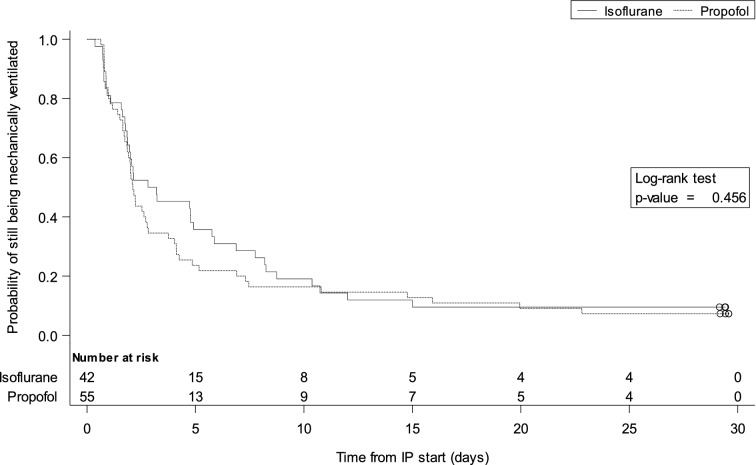
Duration of first mechanical ventilation episode in the 30 days after randomization. IP = investigational product

We found no between-group differences in 30-day mortality (Fig. 2), ventilator-free days alive during the first 30 days (15.8 ± 11.9 with isoflurane vs. 15.9 ± 12.0 with propofol, *p* = 0.931), PaCO_2_ and pH at end of study sedation, time to extubation (Fig. 3) and tidal volume (Table 2).

**Table 2:** Results (whole study population)

Variable (unit)	Timepoint	Isoflurane			Propofol			*p*
		*n*	Mean (± SD)	SM / LSM change from BL	*n*	Mean (± SD)	SM / LSM change from BL	(isoflurane vs. propofol)
PaO_2_/FiO_2_ (mmHg)	BL	72	199 (± 58)		90	202 (± 62)		
	EOT	70	219 (± 76)	0.24/19	89	236 (± 77)	0.46/358	0.185
	D7	41	210 (± 79)	0.03/3	44	185 (± 87)	− 0.15/− 13	0.411
FiO_2_	BL	72	50 (± 14)		90	51 (± 16)		
	EOT	70	46 (± 13)	− 0.28/− 3.4	89	45 (± 12)	− 0.50/− 6.8	0.145
PaCO_2_	BL	71	45.1 (± 9.9)		90	44.6 (± 11.9)		
	EOT	70	47.5(± 10.0)	0.26/2.7	87	45.6 (± 8.6)	0.08/0.9	0.264
pH	BL	71	7.4 (± 0.09)		90	7.4 (± 0.09)		
	EOT	44	7.4 (± 0.08)	0.04/0.00	50	7.4 (± 0.08)	0.17/0.02	0.458
V_T_ (ml/kg)	BL	72	6.6 (± 2.3)		88	6.6 (± 2.0)		
	EOT	56	6,4 (± 2,3)	0.15/0.3	61	6,6 (± 2.1)	0.16/0.3	0.972
Time to extubation	EOT-extubation	42	38 patients extubated		55	51 patients extubated		0.456
30-day mortality	BL-30 days	61	19 patients died		80	19 patients died		0.274
ventilator-free days alive	BL-30 days	59	15.8 (± 11.9)		75	15.9 (± 12.0)		0.931
Ventilator mode	BL	72	Fully controlled: 55.6% Partially assisted: 20.8% Fully assisted: 23.6%		90	Fully controlled: 60% Partially assisted: 25.6% Fully assisted: 14.4%		
	EOT	70	Fully controlled: 38.6% Partially assisted: 21.4% Fully assisted: 40%		89	Fully controlled: 46.1% Partially assisted: 22.5% Fully assisted: 31.5%		

Change in PaO_2_/FiO_2_ ratio between baseline (BL) and end of study sedation (EOT, usually after 48 h)

*LSM*  least squares mean, *SD*  standard deviation, *PaO*_*2*_  arterial partial pressure of oxygen, *FiO*_*2*_ inspired fraction of oxygen, *PaCO*_*2*_  arterial partial pressure of carbon dioxide. *D7*  day seven after randomization. *SM*  standardized means  mean divided by total pooled standard deviation. *V*_*T*_  tidal volume

The odds ratio for successfully achieving assisted spontaneous breathing with isoflurane was 1.596 (95% CI 0.880–2.897; *p* = 0.123) and 1.382 (95% CI 0.690–2.768, *p* = 0.358) for the first and second 24 h of study treatment, respectively.

In the subgroup of patients with PaO_2_/FiO_2_ ≤ 200 mmHg, the increase in PaO_2_/FiO_2_ ratio between baseline and end of study sedation was 152 ± 33 to 186 ± 54 mmHg with isoflurane, and 150 ± 38 to 214 ± 85 mmHg with propofol (*p* = 0.029). At day seven after randomization, PaO_2_/FiO_2_ was 198 ± 69 mmHg in the isoflurane group and 174 ± 106 mmHg in the propofol group (*p* = 0.933). All other respiratory parameters did not significantly differ between both groups as shown in Table 3.

**Table 3:** Results (subgroup with PaO_2_/FiO_2_ < 200 mmHg)

Variable (unit)	Timepoint	Isoflurane			Propofol			*p*
		*n*	Mean (± SD)	SM/LSM change from BL	*n*	Mean (± SD)	SM/LSM change from BL	(isoflurane vs. propofol)
PaO_2_/FiO_2_ (mmHg)	BL	37	152 (± 33)		45	150 (± 38)		
	EOT	36	186 (± 54)	0.43/30	45	214 (± 85)	0.84/66	0.029
	D7	20	198 (± 69)	0.43/36	20	174 (± 106)	0.42/33	0.933
FiO_2_	BL	37	57 (± 14)		45	58 (± 17)		
	EOT	37	51 (± 13)	− 0.34/− 4.6	45	47 (± 11)	− 0.60/− 9.9	0.157
PaCO_2_	BL	37	45 (± 10)		45	46 (± 13)		
	EOT	36	47 (± 8)	0.28/3.1	45	45 (± 10	− 0.02/− 0.3	0.186
pH	BL	37	7.4 (± 0.1)		45	7.4 (± 0.1)		
	EOT	36	7.4 (± 0.1)	0.01/− 0.00	43	7.4 (± 0.1)	0.18/0.02	0.424
V_T_ (ml/kg)	BL	37	6.6 (± 2.8)		45	6.0 (± 1.7)		
	EOT	31	6.2 (± 2.4)	0.10/− 0.00	34	6.2 (± 2.3)	0.22/0.4	0.454
Ventilator mode	BL	37	Fully controlled: 51.4%; Partially assisted: 21.6% Fully assisted: 27%		45	Fully controlled: 68.9% Partially assisted: 24.4% Fully assisted: 6.7%		
	EOT	36	Fully controlled: 41.7% Partially assisted: 16.7% Fully assisted: 41.7%		45	Fully controlled: 48.9% Partially assisted: 24.4% Fully assisted: 26.7%		

Change in PaO_2_/FiO_2_ ratio between baseline (BL) and end of study sedation (EOT, usually after 48 h)

*LSM* least squares mean, *SD* standard deviation, *PaO*_*2*_ arterial partial pressure of oxygen, *FiO*_*2*_ inspired fraction of oxygen, *D7* day seven after randomization, *SM* standardized means mean divided by total pooled standard deviation, *V*_*T*_ tidal volume

In this subgroup, the respective odds ratios for successfully achieving assisted spontaneous breathing with isoflurane during the first 24 and second 24 h of study treatment were 1.722 (95% CI 0.729–4.069; *p* = 0.212) and 1.355 (95% CI 0.492–3.735, *p* = 0.552).

## Discussion

There were no significant differences in arterial oxygenation between propofol and isoflurane in patients with baseline PaO_2_/FiO_2_ ≤ 300 mmHg. There were also no significant differences pH, pCO_2_, V_T_, mortality and ventilator-free days. In the subgroup of patients with baseline PaO_2_/FiO_2_ ≤ 200 mmHg, however, there was a significantly lower PaO_2_/FiO_2_ ratio with isoflurane as compared to propofol. During the 7-day follow-up, no significant differences in oxygenation were observed in the study population as well as the subgroup with baseline PaO_2_/FiO_2_ ≤ 200 mmHg. This observed difference in oxygenation subsided during the first 5 days after the end of study treatment.

The findings are in contrast to the ones by Jabaudon et al., who found better oxygenation and lower levels of sRAGE (a marker of endothelial damage) in ARDS patients sedated with sevoflurane in comparison to midazolam [13]. Multiple possible reasons for this have to be taken into account. First of all Jabaudon et al. were using sevoflurane in contrast to isoflurane in our study. Most previous investigations demonstrating lung-protective effects of volatile anaesthetics were conducted with sevoflurane [13, 15, 16]. Sevoflurane is the only volatile agent that is transformed in a phase II-reaction in about 3–5% into hexafluoro-2-propanol (HFIP) and fluoride. Especially HFIP has shown immunomodulatory effects in both in vitro and animal studies from Urner et al. [17]. These effects have not been demonstrated for isoflurane and its metabolites. Laboratory studies have shown that sevoflurane alters the lipopolysaccharide-induced inflammatory response by downregulation of chemokines and adhesion molecules [18] and decreases the inflammatory response of alveolar macrophages [19].

Another reason for the differing results could be the etiologies and severity of ARDS/AHRF. At least 80% of the patients included in the study by Jabaudon et al. [13] suffered from pneumonia, with a significantly lower PaO_2_/FiO_2_ ratio at inclusion in contrast to our patients. Additionally, our SAPS-II was almost 10 points lower demonstrating a reduced severity of illness in our population.

In an animal study investigating the effects of isoflurane and sevoflurane in pulmonary and extrapulmonary ARDS, Araújo and co-workers found that in pulmonary, but not in extrapulmonary ARDS, sevoflurane reduced interleukin-6 expression, improved respiratory mechanics, led to increased expression of regulators protecting against oxidative stress and epithelial damage and reduced alveolar collapse to a greater degree than isoflurane [20]. This could partly explain our results because it can be assumed that a significant proportion of patients included in our substudy suffered from pulmonary ARDS.

In summary, our results imply that inhaled sedation with isoflurane may not have the same pulmonary protective effects as sevoflurane.

Another plausible explanation for our results is that the lung-protective effects of possible effects of isoflurane inhalation sedation were offset by adverse effects of isoflurane on hypoxic pulmonary vasoconstriction. In comparison to different types of volatile anaesthetics, reductions in shunt volume and increases in oxygenation have been demonstrated for various intravenous sedatives including propofol, diazepam and sodium thiopental [21–23]. This is in line with the results of a study conducted by Abe et al., where propofol improved oxygenation and decreased shunt fraction during one-lung ventilation compared with volatile anaesthetics [24].

## Strengths

This substudy investigated a comparatively large sample of 162 patients with AHRF randomized to isoflurane or propofol. The partial post hoc power analysis revealed the power of the study for detecting statistical significance of a difference in PaO_2_/FiO_2_ ratio of 40 mmHg (as observed by Jabaudon et al. [12]) to be above 90%. Baseline patient characteristics as well as ventilator settings were well-balanced between groups. In addition to the official registration of the Sedaconda trial in the EudraCT database, the present substudy was registered in the German Clinical Trials Registry before opening the study database, minimizing the risk for data selection bias.

## Limitations

This study has certain limitations. As this was a substudy of a larger randomized controlled trial, no a priori sample size calculation could be performed. Even though the partial post hoc power analysis revealed the power of the study for detecting statistical significance of a difference in PaO_2_/FiO_2_ ratio of 40 mmHg between isoflurane and propofol to be 0.901, this substudy may be underpowered for detecting smaller differences in PaO_2_/FiO_2_. In contrast to the study of Jabaudon et al. [13], plasma levels of sRAGE or other markers of endothelial injury were not assessed in our study. Therefore, we had to rely on changes in oxygenation as primary endpoint, a potentially weak surrogate for epi- or endothelial damage that may have been confounded by the possibly opposing effects of isoflurane on hypoxic pulmonary vasoconstriction. The duration of study drug administration was relatively short and the exposure time to isoflurane may have been too short to lead to a clinically manifest reduction in ventilator-induced lung injury. Important ventilation parameters such as respiratory system compliance, airway resistance and presence or absence of auto-PEEP could not be evaluated for the present substudy, as they were not recorded in the main Sedaconda study.

As chest X-ray data were not available for this substudy, we were unable to determine the exact number of patients who met ARDS criteria as defined by the Berlin definition. Even though AHRF due to pneumonia with unilateral opacities or due to congestive heart failure carries a similar prognosis [4], it may still differ from ARDS in pathophysiology. Therefore, a study including only ARDS patients might have resulted in different findings. Moreover, we cannot exclude the possibility that some patients with chronic respiratory impairment may have been included in the present substudy.

## Conclusions

In conclusion, the results show that in patients with ARDS or AHRF, inhaled sedation with isoflurane for a duration of up to 48 h does not lead to improved oxygenation in comparison to intravenous sedation with propofol.

## Data Availability

All data generated or analysed during this study are included in this published article (and its supplementary information files).

## Abbreviations

ABG: Arterial blood gas
ARDS: Acute respiratory distress syndrome
ΔP: Driving pressure
FiO2: Inspired fraction of oxygen
HFIP: Hexafluoro-2-propanol
HPV: Hypoxic pulmonary vasoconstriction
HR: Heart rate
ICU: Intensive care unit
PaCO2: Arterial partial pressure of carbon dioxide
PaO2: Arterial partial pressure of oxygen
PEEP: Positive end-expiratory pressure
RR: Respiratory rate
VT: Tidal volume
